# 

**Published:** 1890-04

**Authors:** 


### International Dental Journal

A MONTHLY PERIODICAL

DEVOTED TO

### DENTAL AND ORAL SCIENCE.

**W. XAVIER SUDDUTH, M.D., D.D.S.,**
**EDITOR.**
**Office, 716 Filbert Street, Philadelphia.**

PUBLISHED BY THE

INTERNATIONAL DENTAL PUBLICATION COMPANY,

**51 WEST THIRTY-SEVENTH STREET, NEW YORK CITY,**

—AND—

**716 FILBERT STREET, PHILADELPHIA.**

ENTERED AT PHILADELPHIA POST-OFFICE AS SECOND-CLASS READING MATTER.

$2.50 per Annum, in Advance. Single Copies, 25 Cents.

PRINTED BY J. B. LIPPINCOTT COMPANY, PHILADELPHIA.

